# Innovative thinking of clinical investigation for rare disease drug development

**DOI:** 10.1186/s13023-023-02909-w

**Published:** 2023-09-22

**Authors:** Peijin Wang, Shein-Chung Chow

**Affiliations:** Department of Biostatistics and Bioinformatics, Duke University School of Medicine, Durham, NC USA

**Keywords:** Composite hypotheses, Demonstrating effectiveness and safety, Real-world data (RWD), Orphan drug development

## Abstract

For the development of a test treatment or drug product, it is necessary to conduct composite hypothesis testing to test for effectiveness and safety simultaneously, since some approved drug products have been recalled due to safety concerns. One of the major issues in conducting a composite hypothesis testing for effectiveness and safety is the requirement of a huge sample size to achieve the desired power for detecting clinically meaningful differences in both safety and effectiveness. Situation can be much difficult in orphan drug development. In this article, a generalized two-stage innovative approach to test for effectiveness and safety simultaneously is proposed. Additionally, to alleviate the requirement of a large randomized clinical trial (RCT) and revealing effectiveness, real-world data is suggested to use in conjunction with RCT data for orphan drug development. The proposed approach can help investigators test for effectiveness and safety at the same time without worrying about the sample size. It also helps reduce the probability of approving a drug product with safety concerns.

## Introduction

Real-world data (RWD) is defined as “data relating to patient health status and/or the delivery of health care that is routinely collected from a variety of sources” [1]. Evidence generated from RWD is called real-world evidence (RWE). RWD/RWE have been frequently used in regulatory submission in terms of evaluating drug effectiveness, safety and benefit-risk balance recently [2, 3]. RWD/RWE can be used for rare disease drug development, historical control in single-arm clinical trials, and post-market effectiveness and safety assessment [2, 4, 5].

For regulatory approval and post-marketing assessment of a new drug (or a new treatment), various types of clinical trials aiming to evaluate the effectiveness and/or safety of the test drug can be considered. Though in Phase I and II trials, safety issue was one of the primary objectives, the sample size of these trials is always small, and only short-term safety issues are considered. As for Phase III trials, though the sample size is larger and the follow-up period is longer, the trials’ primary objective is often efficacy (only using RCT data) or effectiveness (using RWD), i.e., the power for safety objective (secondary objective) may not reach the desired level [6]. This usual practice has caused some approved drug products to be withdrawn after several months or years of approval due to safety concerns [7, 8]. Table 1 lists the top ten unsafe prescribed drugs being withdrawn by FDA between 1993 and 2010. As shown in Table 1, many withdrawn drugs have been used thousands of times per year while on market. This has put patients at unreasonable risk. Similar problem also exists in orphan drug market.

**Table 1:** Top ten prescribed drugs withdrawn by FDA between 1993 and 2010 (Source: Saluja et al.) [8]

Drug name	Use for	Company	Reasons	Years on market	Visit times${^a}$
Rofecoxib	COX-2 selective nonsteroidal anti-inflammatory drug	Merck & Co.	Heart attack and stroke risk	1999–2004	10647
Valdecoxib	Nonsteroidal anti-inflammatory drug	G. D. Searle & Company	Stevens-Johnson syndrome and cardiovascular risk	2001–2005	5612
Cisapride	Gastroprokinetic agent	Janssen Pharmaceuticals	Cardiac toxicity	1993–1999	2196
Troglitazone	Antidiabetic and anti-inflammatory drug	Parke-Davis	Hepatic failure	1997–2000	1976
Gatifloxacin	Antibiotic of the fourth-generation fluoroquinolone family	Kyorin Pharmaceutical Company	Hypoglycemia and hyperglycemia	1999–2003	1332
Tegaserod	A $\text {5-HT}_4$ agonist	Novartis	Cardiovascular risk	2002–2007	980
Cerivastatin	A synthetic lipid-lowering agent	Bayer A.G.	Kidney failure	1997–2001	968
Trovafloxacin	A broad-spectrum antibiotic	Pfizer	Hepatic toxicity	1997–2000	645
Bromfenac	Nonsteroidal anti-inflammatory drug	ISTA Pharmaceuticals	Hepatic failure	1997–1998	434
Mibefradil	Nonselective calcium channel blocker	Roche	Drug interactions	1997–1998	409

$^{a}$ Average number of visits per year during which drug was prescribed

In a post-marketing safety study on orphan drugs, Fan et al. [9] reported that about 69.2% of approved orphan drugs had at least one post-marketing safety event during a 6.74-year follow-up, from 1999 to 2018. And about 1/5 of European Medicines Agency (EMA)-approved orphan drugs either have no benefits or do harm [10]. It is necessary to evaluate both effectiveness and safety at a desired statistical power, instead of treating safety issues as a secondary objective. However, in practice, the typical approach is to power the study based on effectiveness alone and then assess the safety parameters for tolerability. Use composite hypothesis testing to power on both effectiveness and safety is another option, which can alleviate the probability of having an effective but unsafe drug.

Although composite hypothesis testing has the advantage in testing multiple endpoints (e.g., effectiveness endpoint and safety endpoint), many complex issues require full understanding and attention. While utilizing composite hypothesis testing, one intuitive concern would be the required sample size. Composite hypothesis testing always requires a much larger sample size compared with single hypothesis testing [6]. Sample size is always a big worry in typical drug development, let alone orphan disease drug development [11]. Defined by Orphan Drug Act (1983), orphan disease is a disease or condition that affects less than 200,000 people in the US or has a prevalence of less than 7.5 per 10,000 Americans [12]. In other words, orphan disease patients are scattered all over the country with a very limited amount in each medical center. One major problems discussed in this paper about orphan drug development is how to deal with limited and scattered target population, and how to evaluate effectiveness as well as safety simultaneously.

One possible solution to the above-raised problems is RWD. One of the most important properties of RWD compared with RCT data is that it is more accessible and reveals more information about the test product, i.e., it covers a broader range of population with a longer follow-up period [2, 13]. Though it is hard to recruit enough participants for a rare-disease RCT, it is easier to gather RWD about this specific disease all over the country. In this way, the desired statistical power may be achieved.

Resolving these problems not only benefit pharmaceutical companies in developing orphan drugs but also provide valuable clinical evidence of orphan drugs to health technology assessment (HTA) agencies. Specifically, it can assist HTA agencies in evaluating orphan drugs and determining public funding reimbursement. For pharmaceutical manufacturers, the small number of target patients makes it hard for them to recover the drug development cost [14, 15], which makes reimbursement an essential incentive for orphan drug development. Due to economic concerns, oncology orphan drugs have received greater attention compared with non-oncology orphan drugs by investors, since oncology orphan drugs often charge higher prices [16]. Hence, many orphan drugs, especially non-oncology orphan drugs and ultra-orphan drugs, eagerly need reimbursement to incent development.

Though reimbursement may greatly enhance orphan drug development, currently, the probability of negative HTA recommendation is high. In some Europe countries, the negative recommendation probability was about 30–40%; and only about 20–25% of recommended orphan drugs that truly obtained reimbursement [17, 18]. One commonly used framework to evaluate orphan drugs is multi-criteria decision analysis (MCDA). Well-accepted criteria in MCDA are comparative effectiveness/efficacy, existence of alternatives, disease severity, safety, target population size and quality of evidence [18–20]. However, there is still disagreement on how to select criteria, how to quantify factors, and how to determine the weight for each criterion [14]. One way to improve recommendation probability is to provide more reliable information about the effectiveness and safety of the orphan drug to HTA.

In this article, some innovative thinking regarding composite hypothesis testing for effectiveness and safety is proposed. These out-of-box innovative thinkings include (i) utilizing RWD in combination with RCT data to demonstrate effectiveness and safety; (ii) determining null and corresponding alternative hypotheses of composite hypothesis testing; and (iii) proposing a generalized two-stage approach to conduct composite hypothesis testing with superiority test(s).

In the next section, challenging issues regarding RWD are presented. Detailed innovative thinking about composite hypothesis testing is illustrated in “Composite hypothesis testing for effectiveness and safety” Section. The generalized two-stage approach for composite hypothesis testing with superiority test(s) is proposed in “The implementation of RWD in composite hypothesis testing” Section. Finally, the concluding remarks are provided in “Concluding Remarks” Section.

## Challenging issues about real-world data

RWD has become a hot topic due to its large quantity and unique nature in assessing the real-world performance of a new drug or a new treatment. Though the idea of using RWD evaluating effectiveness and safety has been proposed for several years, RCT is still the gold standard in this field. A well-controlled RCT has two essential features: randomization and blindness, which can eliminate potential bias and the impact of confounding. However, RCTs also have some limitation, such as (i) RCTs have many restrict inclusion and exclusion criteria, and (ii) RCTs have a very limited study population and follow-up period. In this way, RCTs create a too idea condition to evaluate the real-world performance of the test drug, and it is hard for some trials to recruit enough participants. Thanks to RWD, the real-world performance of the test drug can be determined, information for a broader study population can be collected, and the follow-up period can also be longer. Thus, RWD can be very helpful in drug development, especially for orphan drugs [2].

Though RWD could become a complement to RCT data, there are many concerns about how to utilize RWD in drug development. For instance, the biased nature of RWD, i.e., without randomization, the treatment difference detected from RWD might be because of confounding. Additionally, without blindness, whether the patient receives the test treatment is subjective, and patients may be more likely to drop off if the treatment is not beneficial. Due to these unfavorable characteristics of RWD, it is essential to pay attention to how the data were collected and the methodology used to conduct the research [21]. In this section, challenging issues regarding the use of RWD in support of clinical investigation of the test treatment are demonstrated.

### Representativeness of RWD

In a clinical trial evaluating the performance of a test drug for a given disease, it is essential to determine whether RWD is representative of the target population because RWD are usually collected from different individual studies and might have (i) different structural or nonstructural format, (ii) similar but different study protocols, (iii) similar but different inclusion/exclusion criteria, (iv) similar but different target populations, (v) similar but different study objectives/hypothesis/endpoints, (vi) similar but different trial procedures, and (vii) similar but different statistical procedures; or electronic health records, which makes it necessary to assess patients’ geography, health status and received health care [22]. It is necessary to generate some approaches to test for the representativeness of RWD; however, currently, there is no generally accepted method to test for representativeness.

### Heterogeneity of RWD

Heterogeneity of RWD is another essential issue that needs to be considered before using RWD in practice, which exists due to the difference within and across individual studies with different means, variances, and sample sizes. The treatment-by-study interaction among individual studies also can affect the poolability for final analysis. Detailed information on how to evaluate the heterogeneity of RWD can be found in Moran et al. [23].

### Confounding/interaction of RWD

Confounding exists in RWD because of the difference in baseline demographics, such as age, gender, weight/height or BMI, race, etc., and patient characteristics, such as disease severity, medical history, concomitant medication, etc. Yang et al. [24] proposed to use the confounding function to summarize the impact of unobserved confounders on outcome variables while accounting for observed covariates to improve the inference based on RWD. As for interaction, it exists may be because of different treatments, centers, and covariates (demographics and patient characteristics). Data should not be pooled for analysis if a significant qualitative interaction is observed. Data may be pooled for final analysis though a significant quantitative interaction is observed.

### Missing data of RWD

Missing data or incomplete data are commonly encountered in biomedical research, because of dropouts, loss of follow-up, withdraw of informed consent, withdraw by investigators, etc. Missingness could also exist due to non-medical reasons, such as health insurance plan issues. It is important to first determine whether the loss of follow-up or other missingness will significantly affect study conclusions. If there is no significant influence, then missingness is less critical [22]. Otherwise, a proper approach to handling missing data or incomplete data should be considered.

### Reproducibility/generalizability of RWD

Reproducibility means the observed clinical result using RWD at one study center can also be found at another study center, where the target population remains the same. Shao and Chow [25] have proposed to use reproducibility probability for a given clinical trial to evaluate reproducibility. They have considered three approaches to analyze reproducibility probability, including the estimated power approach, confidence bound approach, and Bayesian approach. As for generalizability, it suggests that the clinical results from one target population (e.g., adults) can be generalized to another similar but different target patient population (e.g., children or elderly). The generalizability can be evaluated using the sensitivity index proposed by Chow [26].

### Data quality and validity of RWD

While using RWD to conduct a study on drug development, we should also pay attention to the confidentiality agreement for data sharing and the development of standard forms of data capture. As for the data management process, many essential issues should be paid attention to, such as data transfer, data review/query, data verification/validation, and database lock. Gliklich and Leavy [27] proposed several criteria evaluating data quality for RWD to ensure the reliability of real-world evidence.

## Composite hypothesis testing for effectiveness and safety

Traditionally, the effectiveness of a test drug was analyzed first by selecting a proper sample size for achieving the desired power in detecting treatment effect. Using the same data, some approaches are considered to determine whether there is safety concern. However, this idea is unfavorable since the study is not powered to test for safety. The sample size required to test for safety is often larger than the one for effectiveness, since the adverse event incidence rate difference between treatment and control is much lower compared with typical effectiveness assessment. According to Chow et al. [28], the sample size of extremely low incidence rate trials can be very large. Hence, it is inappropriate to establish a study based on the effectiveness endpoint alone and evaluate both effectiveness and safety at the same time since the power for evaluating safety may below the desired level. To avoid this from happening, composite hypothesis testing for effectiveness and safety in regulatory approval is one of the solutions.

### Innovative thinking for composite hypothesis testing

For testing composite hypotheses for both effectiveness and safety, the first step is to determine the null and alternative hypotheses. There are nine possible combinations of composite hypotheses while considering effectiveness and safety at the same time. Table 2 lists all nine possible combinations. The first letter of each combination representing the type of hypothesis testing for effectiveness and the second letter representing the type of hypothesis testing for safety. For instance, “SN” represents superiority test in effectiveness and non-inferiority test in safety, and the composite hypotheses can be written as

1 $$\begin{gathered} H_{0} {\text{: no superiority in effectiveness or inferiority in safety or both v}}{\text{.s}}{\text{. }} \hfill \\ H_{A} {\text{: superiority in effectiveness and non - inferiority in safety}}{\text{.}} \hfill \\ \end{gathered}$$

**Table 2:** Possible composite hypotheses of testing for effectiveness and safety

	Safety		
	N^a^	S^b^	E^c^
Effectiveness			
N	NN	NS	NE
S	SN	SS	SE
E	EN	ES	EE

^a^ N = non-inferiority

^b^ S = superiority

^c^ E = equivalence

Superiority test requires a larger sample size compared with non-inferiority test. The sample size calculation formula of control arm for non-inferiority test and superiority test are:

$$\begin{aligned} \frac{(z_{1-\alpha }+z_{1-\beta })^2}{(p_1-p_2+\delta )^2} \left[ \frac{p_1(1-p_1)}{k}+p_2(1-p_2)\right] , \end{aligned}$$

and

$$\begin{aligned} \frac{(z_{1-\alpha }+z_{1-\beta })^2}{(p_1-p_2-\delta )^2} \left[ \frac{p_1(1-p_1)}{k}+p_2(1-p_2)\right] , \end{aligned}$$

respectively, where $p_1-p_2$ is the incidence rate difference of adverse events, *k* is the allocation ratio, and $\delta$ is the non-inferiority/superiority margin ($\delta >0$). The difference between these two formulae is the denominator of the first term. The denominator of superiority tests is smaller, which makes the sample size larger. And composite hypothesis testing requires a larger sample size compared with single hypothesis testing. Therefore, a composite hypothesis testing with superiority test(s) requires a very large sample size to maintain enough power.

To make good use of all the data, the two-stage innovative approach proposed by Chow [29] is generalized for composite hypothesis testing with superiority test(s). Before discussing the details of the proposed generalized two-stage approach, first mention the difference between superiority and non-inferiority tests.

**Fig. 1:**
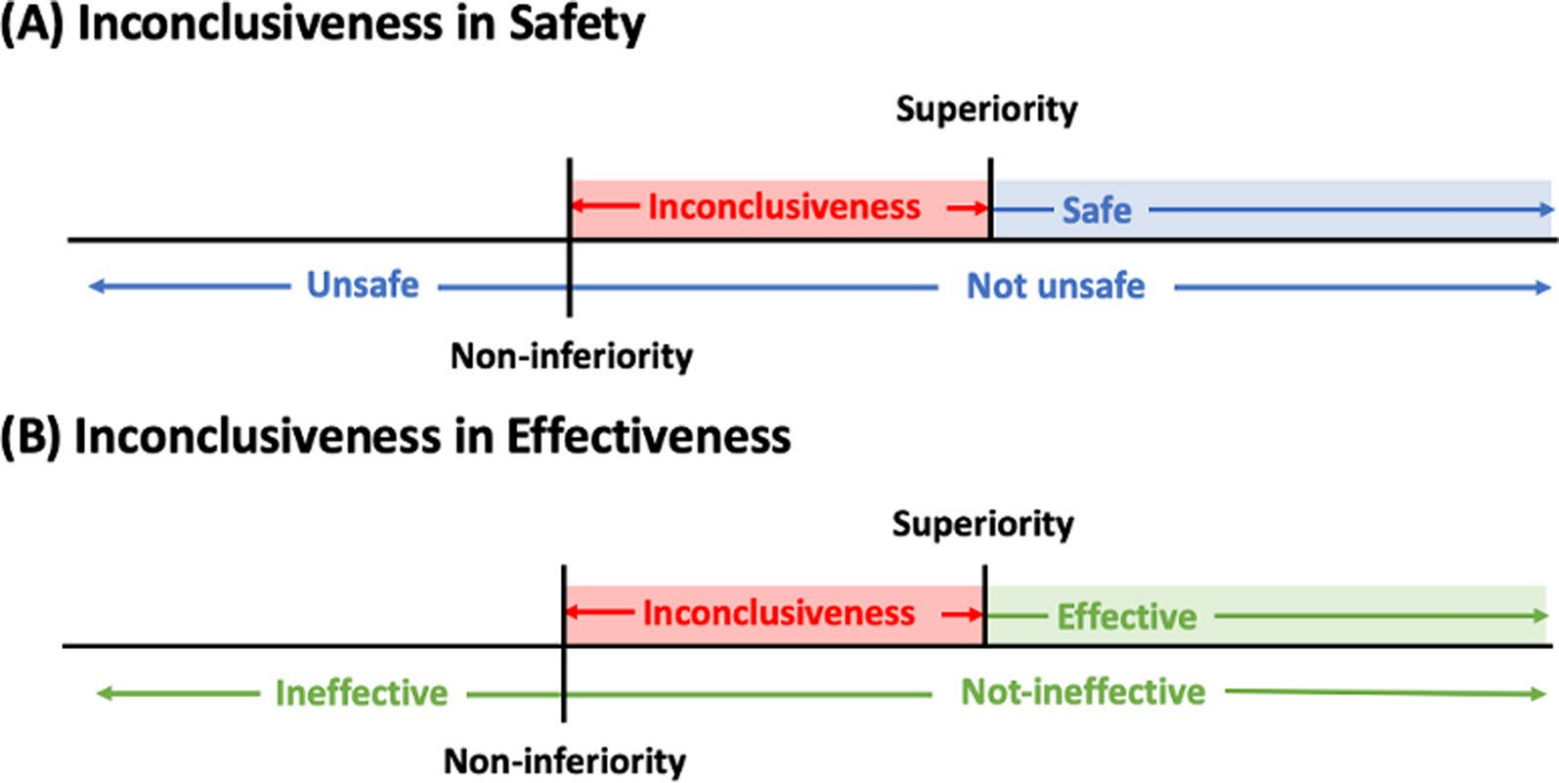
Illustration of inconclusiveness in safety and effectiveness

While conducting a non-inferiority test for safety, the alternative hypothesis ($H_A$) is “not-unsafe”; while the $H_A$ of a superiority test is “safe”. As shown in Fig. 1A, there would be an inconclusiveness zone between “not-unsafe” and “safe”. If the primary objective is to test for safety (superiority test), instead of directly conducting this superiority test, another option is to conduct the non-inferiority test first demonstrating the “not-unsafety” of the test drug. If the null hypothesis ($H_0$) of non-inferiority test is rejected, then test for the probability of inconclusiveness. If the probability of inconclusiveness is negligible, we may finally conclude the safety of the test drug [29]. Similarly, there is an inconclusiveness zone between “effectiveness” and “not-ineffectiveness” as shown in Fig. 1B. The main advantage of using this approach is that it allows us to stop early for inferiority in effectiveness and/or safety.

### Conduct hypothesis testing

**Fig. 2:**
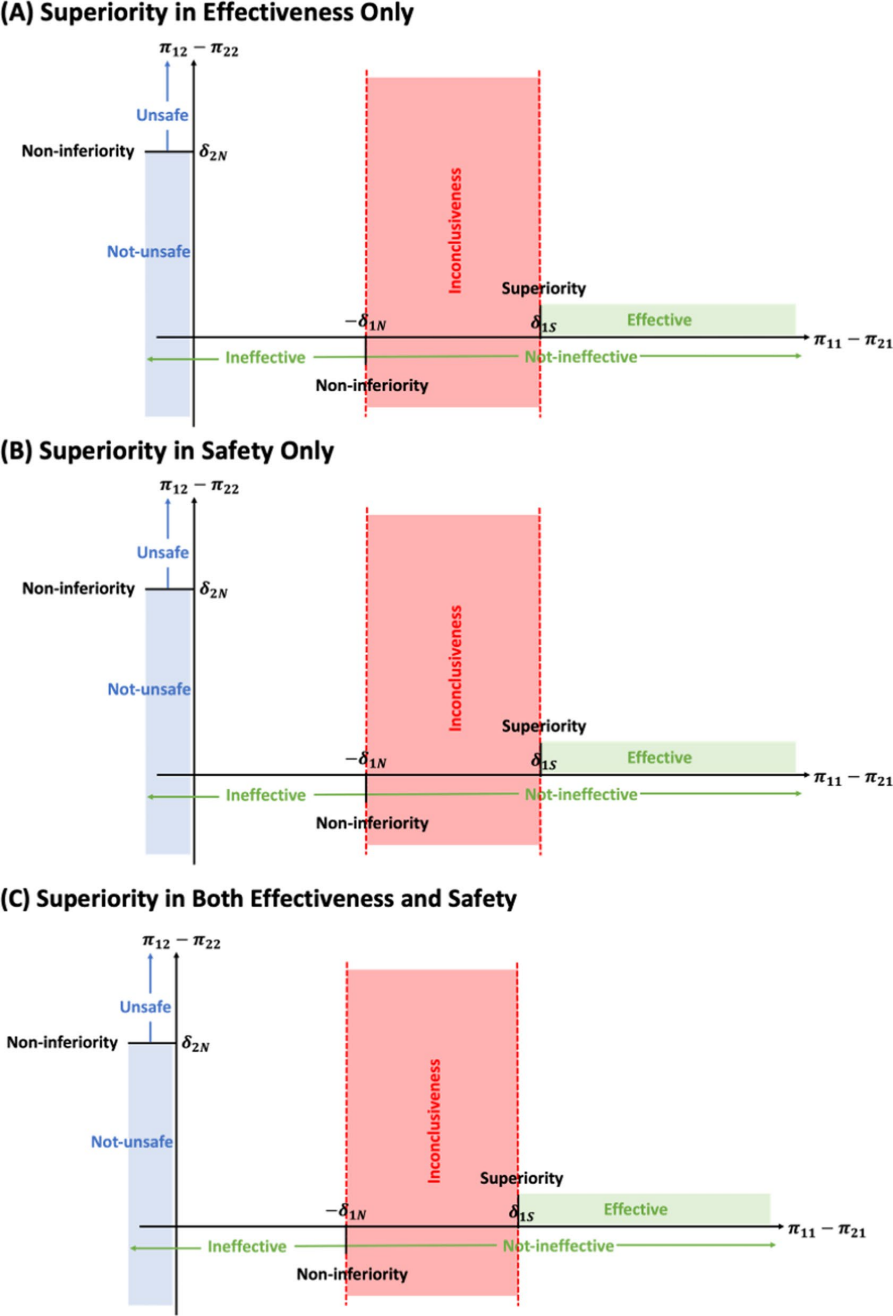
Demonstration of composite hypothesis testing with superiority test(s)

There are three types composite hypothesis testing that are applicable for the proposed two stage approach: (i) “SN” (superiority test for effectiveness and non-inferiority test for safety), (ii) “NS” (non-inferiority test for effectiveness and superiority test for safety), and (iii) “SS” (superiority test for effectiveness and safety). In composite hypothesis testings, the definition of inconclusiveness also exist, details are shown in Fig. 2. Specifically, for "SN" hypothesis testing (in Fig. 2(A)), the inconclusiveness exists due to effectiveness only; for "NS" hypothesis testing (in Fig. 2(B)), the inconlusiveness exists due to safety only; and for "SS" hypothesis testing (in Fig. 2(C)), both effectiveness and safety will lead to inconclusiveness. Here, use “SN” as an example to illustrate how to set up hypothesis, derive test statistic and calculating the required sample size to reach desired statistical power.

Let $X_{ijk}$ denote the response of binary outcome *k* from the *j*th subject in the *i*th arm, where $i\in \{1,2\}$ represent the treatment and control arm, $j\in \{1,\cdots ,n_i\}$, and $k\in \{1,2\}$ represent effectiveness and safety endpoint. $X_{ijk}\sim \text {Bernoulli}(\pi _{ik})$. Specifically, $X_{ij1}=1$ represents that the patient was benefit from the test treatment, and $X_{ij2}=1$ represents that the patient experienced adverse events. For simplicity and illustration purpose, assume the allocation ratio is 1 : 1, i.e., $n_1=n_2=n$. The hypotheses for Stage 1 (non-inferiority tests) are as follows:

2 $$\begin{gathered} H_{0} :\pi _{{11}} - \pi _{{21}} \le - \delta _{{1N}} {\text{ or }}\pi _{{12}} - \pi _{{22}} \ge \delta _{{2N}} {\text{ or both v}}{\text{.s}}{\text{.}} \hfill \\ H_{A} :\pi _{{11}} - \pi _{{21}} > - \delta _{{1N}} {\text{ and }}\pi _{{12}} - \pi _{{22}} < \delta _{{2N}} , \hfill \\ \end{gathered}$$

where $\delta _{1N}$ and $\delta _{2N}$ are the non-inferiority for effectiveness and safety, respectively. The test statistic for hypothesis testing shown in Eq. (2) is

3 $$\begin{aligned} Z_1 = \frac{p_{11}-p_{21}+\delta _{1N}}{se_{10}} \text { and } Z_2 = \frac{p_{12}-p_{22}-\delta _{2N}}{se_{20}}, \end{aligned}$$

where $se_{k0}=\sqrt{\frac{2}{n}p_k(1-p_k)}$, $p_k=\frac{p_{1k}+p_{2k}}{2}$, $p_{ik}=\frac{\sum _j X_{ijk}}{n}$. Using the asymptotic normal method without continuity correction proposed by Sozu et al. [30], the asymptotic statistical power can be derived as (detailed derivation is shown in Appendix A1):

4 $$\begin{aligned} \text {Power}&= \, \Phi \left( \frac{(p_{11}-p_{21}+\delta _{1N})-se_{10}z_{1-\alpha }}{se_1}\right) \\&\quad - \Phi _\text {nml}\left( \frac{(p_{11}-p_{21}+\delta _{1N})-se_{10}z_{1-\alpha }}{se_1},\frac{(p_{12}-p_{22}-\delta _{2N})-se_{20}z_{\alpha }}{se_2}\right) , \end{aligned}$$

where $\Phi (\cdot )$ is the cumulative distribution function (CDF) for standard normal distribution, and $\Phi _\text {nml}$ is the CDF for 2-variate standard normal distribution. Applying power analysis method, the sample size for composite hypothesis testing can be determined iteratively [30]:

*Step 1*: Specify Bernoulli distribution parameters $\pi _{ik}$ ($i,k\in \{1,2\}$), the correlations between two outcome variables, and the non-inferiority margins.
*Step 2*: Set initial value of $n^{(0)}$ and compute the statistical power $(1-\beta )^{(0)}$.
*Step 3*: Repeat **Step 2** by gradually increasing the sample size, until the statistical power reaches the desired level.

The hypotheses for Stage 2 (test for inconclusiveness) are

5 $$\begin{gathered} H_{0} :P_{I} ( - \delta _{{1N}} < \pi _{{11}} - \pi _{{21}} < \delta _{{1S}} ) \ge p_{0} {\text{ v}}{\text{.s}}{\text{.}} \hfill \\ H_{A} :P_{I} ( - \delta _{{1N}} < \pi _{{11}} - \pi _{{21}} < \delta _{{1S}} ) < p_{0} , \hfill \\ \end{gathered}$$

where $P_I$ stands for the probability of inconclusiveness, $\delta _{1S}$ is the superiority margin for effectiveness, and $p_0$ is the pre-determined threshold for $P_I$. The test statistic derived under $H_0$ in Eq. (5) is

6 $$\begin{aligned} Z=\frac{{\hat{P}}_I-p_0}{\sqrt{p_0(1-p_0)/m}}\sim N(0,1), \end{aligned}$$

where the calculation of ${\hat{P}}_I$ is shown in Appendix A2, and *m* is the sample size. The approximate statistical power is

7 $$\begin{aligned} \Phi \left( \frac{\sqrt{m}(p_0-P_I)-z_{\alpha } \sqrt{p_0 (1-p_0)}}{\sqrt{p_0(1-p_0)}}\right) , \end{aligned}$$

where $P_I$ is the true probability of inconclusiveness under $H_A$ in Eq. (5), and $z_q$ is the *q*th quantile of standard normal distribution. The sample size m can be computed using Eq. (8):

8 $$\begin{aligned} m=\frac{(z_{1-\beta }+z_\alpha )^2 p_0 (1-p_0)}{(p_0-P_I)^2}. \end{aligned}$$

### Simulation for composite hypothesis testing

One major goal of this paper is to emphasize the importance of RWD/RWE in orphan drug development. A simulation is conducted aiming to compare the required sample size for composite hypothesis testing for Stage 1 to a typical non-inferiority test for effectiveness. As state earlier, if a study sets the effectiveness as the primary objective whereas safety as the secondary objective, there will be no enough power to test for safety. Table 3 presents the sample sizes for the composite hypothesis testing in Eq. (2) and single hypothesis testing for effectiveness only.

Fixing $\pi _{11}$, $\pi _{21}$ and $\delta _{1N}$, the sample size of the composite hypothesis testing is more than 3 times larger than the sample size of single hypothesis testing, especially when $\pi _{12}$ and $\pi _{22}$ are close, e.g., when $\pi _{12}=0.07$ and $\pi _{22}=0.08$, the sample size for composite test is around 9000, whereas the one for effectiveness test is only around 300. This suggests that while using effectiveness as primary objective, the power for secondary objective (safety) is very limit. The required sample size for composite hypothesis testing is extremely large, which makes it impossible for orphan drug trials. Though the composite test’s sample size is associated with the non-inferiority margin for safety, the overall sample size is still more than 700. Thus, it is necessary to utilize RWD in orphan drug development; otherwise, it is difficult to maintain enough power for testing for effectiveness and safety.

**Table 3:** Sample size comparison of composite hypothesis testing and single hypothesis testing

Effectiveness		Safety		$N_{\text {com}}^a$	$N_{\text {eff}}^b$
Treatment ($\pi _{11}$)	Control ($\pi _{21}$)	Treatment ($\pi _{12}$)	Control ($\pi _{22}$)		
$$\delta _{1N}=0.1, \delta _{2N}=0.001.$$					
0.6	0.5	0.05	0.08	992	385
		0.06		2317	
		0.07		9000	
0.7	0.5	0.05	0.08	992	362
		0.06		2317	
		0.07		9000	
0.8	0.5	0.05	0.08	992	322
		0.06		2317	
		0.07		9000	
$$\delta _{1N}=0.1, \delta _{2N}=0.005.$$					
0.6	0.5	0.05	0.08	778	385
		0.06		1635	
		0.07		4840	
0.7	0.5	0.05	0.08	778	362
		0.06		1635	
		0.07		4840	
0.8	0.5	0.05	0.08	778	322
		0.06		1635	
		0.07		4840	
$$\delta _{1N}=0.3, \delta _{2N}=0.001.$$					
0.6	0.5	0.05	0.08	992	43
		0.06		2317	
		0.07		9000	
0.7	0.5	0.05	0.08	992	41
		0.06		2317	
		0.07		9000	
0.8	0.5	0.05	0.08	992	36
		0.06		2317	
		0.07		9000	

$^{a}$ $N_\text {com}$ = sample size for composite hypothesis testing for effectiveness and safety

$^{b}$ $N_\text {eff}$ = sample size for single hypothesis testing for effectiveness

Correlation between effectiveness and safety are assumed to be the same for treatment and control group, which is $\rho _1=\rho _2=0.3$. $\alpha =0.025$ and $1-\beta =0.80$

## The implementation of RWD in composite hypothesis testing

Based on the simulation study, the required sample size for the two-stage approach is extremely large. Following Chow and Huang [13], we propose to use RCT data in conjunction with RWD data to test for the probability of inconclusiveness. Again, use “SN” type of composite hypothesis testing as an example to illustrate the two-stage approach.

The first stage is to test for non-inferiority in both effectiveness and safety. In other words, the first step aims to determine whether the test drug is both “not-ineffective” in effectiveness and “not-unsafe” in safety. After confirming “not-ineffectiveness” and “not-unsafety”, the second step is to use RCT data in conjunction with RWD to eliminate the probability of inconclusiveness for “effectiveness”. If the probability for inconclusiveness is of no or little clinical meaning or importance, we then conclude that the test treatment is superior to the control in terms of effectiveness and non-inferior to the control in terms of safety.

This generalized two-stage innovative approach using RWD for testing “SN” composite hypotheses can be briefly summarized below:

*Step 1*: At the first stage, following Chow et al. [28], derive the required sample size achieving the desired power. With the selected sample size, the composite hypothesis testing, as shown in Eq. (2), will be conducted to test for “not-ineffectiveness” and “not-unsafety”. Note that statistical test will be performed based on data collected from RCT and RWD at the first stage.
*Step 2*: If we fail to reject the null hypothesis in Eq. (2), then stop the trial. Otherwise, we may conclude that the test drug is “not-ineffective” and “not-unsafe” and move to Step 3.
*Step 3*: Compute the sample size required for obtaining desire power in conducting one-sided test for the probability of inconclusiveness. At this step, At this step, new samples from RWD will be used to control type I error rate. However, if the data used in Stage 1 will be continued to use at Stage 2, some approaches to control family-wise type I error rate need to be considered.
*Step 4*: Test hypotheses as shown in Eq. (5). If we fail to reject the null hypothesis for the probability of inconclusiveness, i.e., the probability of inconclusiveness could not be ignored. Then we fail to conclude the superiority of the test treatment in terms of effectiveness. Otherwise, the superiority in terms of effectiveness is confirmed.

In summary, the proposed two-stage innovative approach for composite hypothesis testing would be helpful especially for superiority test(s), i.e., the composite hypotheses contain one or more superiority tests. In this way, the trial could be allowed to stop early and avoid requiring a large RCT.

## Concluding remarks

Many clinical trials for drug development are powered on effectiveness only, and safety issue is considered as the secondary objective. This practice has made some approved drugs have safety concerns, and some are even withdrawn or recalled. One possible reason for researchers not power on safety is that testing for safety requires a much larger sample size. As for orphan drug development, this problem is even worse due to the limited availability of participants. In this article, a generalized two-stage approach to conduct composite hypothesis testing with superiority test(s) for evaluating effectiveness and safety simultaneously is proposed. Building on Chow [29]’s idea, the first stage is to conduct composite non-inferiority tests, and the second stage is to test for the probability of inconclusiveness, after rejecting the null hypothesis in Stage 1. Since composite hypothesis testing with superiority test(s) requires a large sample size, we further proposed to use RCT data in conjunction with RWD to to meet the desired power level.

This idea can greatly benefit orphan drug development. Though the definition of rare disease in different countries are different, it is always difficult to recruit enough patients with the specific rare disease. Using RWD in orphan drug development or assessment has many advantages: (i) revealing real-world performance of the test orphan drug; (ii) allowing to assess effectiveness and safety at the same time with desired power; and (iii) providing more valuable information for reimbursement. In this way, more orphan drug reimbursement may be offered to pharmaceutical manufacturers to incent both non-oncology and oncology orphan drug development. Additionally, RWD may provide more information regarding the performance of the orphan drug than conventional RCTs. For example, RWD allows to assess the drug for a longer follow-up period and broader population. In this way, greater insight for orphan drugs can be provided, which may help reimbursement assessment. It, however, should be noted that the good characteristics of RWD are the key factors for the success of the proposed approach. The good characteristics of RWD include, but are not limited to, representativeness, quality, consistency, and relevancy.

## Data Availability

Data sharing not applicable to this article as no datasets were generated or analysed during the current study.

## Apppendix

### A1: Power calculation for stage 1

The asymptotic power formula is

9 $$\begin{aligned} {\text{Power}} & = P(\{ Z_{1} > z_{{1 - \alpha }} \} \cap \{ Z_{2} \le z_{\alpha } \} |H_{A} {\text{ is true}}{\text{.)}} \\ & = P(\{ Z_{1}^{*} > \frac{{se_{{10}} z_{{1 - \alpha }} - (\pi _{{11}} - \pi _{{21}} + \delta _{{1N}} )}}{{se_{1} }}\} \cap \{ Z_{2}^{*} \le \frac{{se_{{20}} z_{\alpha } - (\pi _{{12}} - \pi _{{22}} - \delta _{{2N}} )}}{{se_{1} }}\} |\pi _{{11}} ,\pi _{{21}} ,\pi _{{12}} ,\pi _{{22}} ), \\ \end{aligned}$$

where $Z_k^*=\frac{p_{1k}-p_{2k}-(\pi _{1k}-\pi _{2k})}{se_k}$, $se_k=\sqrt{\frac{1}{n}(v_{1k}+v_{2k})}$, and $v_{ik}=\pi _{ik}(1-\pi _{ik})$. Since $Z_k^*$ follows standard normal distribution, $\varvec{Z^*}=(Z_1^*,Z_2^*)^T$ follows 2-variate standard normal distribution $N_2({\varvec{0}},\varvec{\Sigma })$, where the diagonal elements are 1s, and off-diagonal elements are [30]

10 $$\begin{aligned} \rho _\text {nml}=\frac{\sum _{i=1}^2\rho _i\sqrt{v_{i1}v_{i2}}}{\sqrt{(v_{11}+v_{21})(v_{12}+v_{22})}}, \end{aligned}$$

where $\rho _i$ is the correlation coefficient between effectiveness and safety for the *i*th arm. Since effectiveness and safety are correlated, using the asymptotic normal method without continuity correction proposed by Sozu et al. [30]. The power in Eq. (9) can be written as

11 $$\begin{gathered} P\left( {\{ Z_{1}^{*} > \frac{{se_{{10}} z_{{1 - \alpha }} - (\pi _{{11}} - \pi _{{21}} + \delta _{{1N}} )}}{{se_{1} }}\} \cap \{ Z_{2}^{*} \le \frac{{se_{{20}} z_{\alpha } - (\pi _{{12}} - \pi _{{22}} - \delta _{{2N}} )}}{{se_{2} }}\} } \right) \hfill \\ = P\left( {Z_{1}^{*} > \frac{{se_{{10}} z_{{1 - \alpha }} - (\pi _{{11}} - \pi _{{21}} + \delta _{{1N}} )}}{{se_{1} }}} \right) \hfill \\ - P\left( {\{ Z_{1}^{*} > \frac{{se_{{10}} z_{{1 - \alpha }} - (\pi _{{11}} - \pi _{{21}} + \delta _{{1N}} )}}{{se_{1} }}\} \cap \{ Z_{2}^{*} > \frac{{se_{{20}} z_{\alpha } - (\pi _{{12}} - \pi _{{22}} - \delta _{{2N}} )}}{{se_{2} }}\} } \right) \hfill \\ = \Phi \left( {\frac{{(p_{{11}} - p_{{21}} + \delta _{{1N}} ) - se_{{10}} z_{{1 - \alpha }} }}{{se_{1} }}} \right) \hfill \\ - \Phi _{{{\text{nml}}}} \left( {\frac{{(p_{{11}} - p_{{21}} + \delta _{{1N}} ) - se_{{10}} z_{{1 - \alpha }} }}{{se_{1} }},\frac{{(p_{{12}} - p_{{22}} - \delta _{{2N}} ) - se_{{20}} z_{\alpha } }}{{se_{2} }}} \right), \hfill \\ \end{gathered}$$

where $\Phi (\cdot )$ is CDF of standard normal distribution, and $\Phi _\text {nml}(\cdot )$ is the CDF of $N_2({\varvec{0}},\varvec{\Sigma })$.

### A2: Probability of inconclusiveness

The probability of inclusiveness $P_I$ is derived as:

12 $$\begin{aligned} P_{I} : & = P_{I} ( - \delta _{{1N}} < \pi _{{11}} - \pi _{{21}} < \delta _{{1S}} ) \\ & = P( - \delta _{{1N}} < \pi _{{11}} - \pi _{{21}} < \delta _{{1S}} |\pi _{{11}} - \pi _{{21}} > - \delta _{{1N}} ) \\ & = \frac{{P( - \delta _{{1N}} < \pi _{{11}} - \pi _{{21}} < \delta _{{1S}} )}}{{P(\pi _{{11}} - \pi _{{21}} > - \delta _{{1N}} )}} = \frac{{P(\{ Z_{1} > z_{{1 - \alpha }} \} \cap \{ Z^{\prime}_{1} > z_{{1 - \alpha }} \} )}}{{P(\{ Z_{1} > z_{{1 - \alpha }} \} )}} \\ & = \frac{{P\left( {\{ Z_{1}^{*} > \frac{{se_{{10}} z_{{1 - \alpha }} - (\pi _{{11}} - \pi _{{21}} + \delta _{{1N}} )}}{{se_{1} }}\} \cap \{ Z_{1}^{*} > \frac{{se_{{10}} z_{{1 - \alpha }} - (\pi _{{11}} - \pi _{{21}} - \delta _{{1S}} )}}{{se_{1} }}\} } \right)}}{{P\left( {\{ Z_{1}^{*} > \frac{{se_{{10}} z_{{1 - \alpha }} - (\pi _{{11}} - \pi _{{21}} + \delta _{{1N}} )}}{{se_{1} }}\} } \right)}} \\ & = \frac{{\Phi \left( {\frac{{(\pi _{{11}} - \pi _{{21}} - \delta _{{1S}} ) - se_{{10}} z_{{1 - \alpha }} }}{{se_{1} }}} \right)}}{{\Phi \left( {\frac{{(\pi _{{11}} - \pi _{{21}} + \delta _{{1N}} ) - se_{{10}} z_{{1 - \alpha }} }}{{se_{1} }}} \right)}}, \\ \end{aligned}$$

where $Z_1'=\frac{p_{11}-p_{21}-\delta _{1S}}{se_{10}}$. Therefore, ${\hat{P}}_I$ can be computed by plugging the observed data.
